# Consensus Statement on the Management of Duchenne Muscular Dystrophy in Saudi Arabia During the Coronavirus Disease 2019 Pandemic

**DOI:** 10.3389/fped.2021.629549

**Published:** 2021-02-17

**Authors:** Ahmed K. Bamaga, Fouad Alghamdi, Nahla Alshaikh, Waleed Altwaijri, Fahad A. Bashiri, Khalid Hundallah, Musaad Abukhaled, Osama Y. Muthaffar, Sameer Al-Mehmadi, Tahani Ahmed Jamaly, Mohammad A. Al-Muhaizea, Abdulaziz Al-Saman

**Affiliations:** ^1^Neurology Division, Pediatric Department, Faculty of Medicine, King Abdulaziz University Hospital, King Abdulaziz University, Jeddah, Saudi Arabia; ^2^Pediatric Department, King Faisal Specialist Hospital and Research Center, Jeddah, Saudi Arabia; ^3^King Fahad Specialist Hospital, Dammam, Saudi Arabia; ^4^Pediatric Department, Ministry of National Guard Health Affairs, Jeddah, Saudi Arabia; ^5^College of Medicine, King Saud Bin Abdulaziz University for Health Sciences, Jeddah, Saudi Arabia; ^6^King Abdullah International Medical Research Center, Jeddah, Saudi Arabia; ^7^Pediatric Department, King Saud Bin Abdulaziz University, Riyadh, Saudi Arabia; ^8^Pediatric Department, King Abdullah Specialized Children Hospital, Riyadh, Saudi Arabia; ^9^Department of Pediatrics, College of Medicine, King Saud University, Riyadh, Saudi Arabia; ^10^Division of Neurology, Department of Pediatrics, King Saud University Medical City, Riyadh, Saudi Arabia; ^11^Prince Sultan Military Medical City, Riyadh, Saudi Arabia; ^12^Department of Neuroscience, King Faisal Specialist Hospital and Research Centre, College of Medicine, Alfaisal University, Riyadh, Saudi Arabia; ^13^Department of Pediatric Neurology, Children's Hospital, King Saud Medical City, Riyadh, Saudi Arabia; ^14^Children Health Center, International Medical Center, Jeddah, Saudi Arabia; ^15^Pediatric Neurology Department, National Neuroscience Institute, King Fahad Medical City, Riyadh, Saudi Arabia

**Keywords:** COVID-19, Duchenne muscular dystrophy, consensus, telemedicine, virtual clinic

## Abstract

**Background:** The coronavirus disease 2019 (COVID-19) pandemic has caused overwhelming challenges in healthcare worldwide. During such an outbreak, some needs of high-risk groups who require regular follow-ups and long-term management are not met. The vulnerable populations include patients with Duchenne muscular dystrophy (DMD). Duchenne muscular dystrophy is characterized by respiratory complications caused by muscle weakness. Hence, patients with this condition are at high risk of severe diseases including COVID-19.

**Methods:** To standardize care and provide optimal treatment to DMD patients in Saudi Arabia during the COVID-19 pandemic, a panel of experts including neurologists and pediatricians consolidated recommendations for healthcare professionals and caregivers.

**Results:** During this pandemic, substituting unnecessary clinic visits with virtual clinic services was highly recommended, if possible, without compromising clinical outcomes. Duchenne muscular dystrophy patients with respiratory complications should be closely monitored, and those with cardiovascular complications must continue taking angiotensin-converting enzyme inhibitors or angiotensin receptor blockers. Moreover, individualized home-based rehabilitation management was preferred. Glucocorticoid and new gene correction therapies should be continued. However, new gene correction therapy must be post-poned in newly diagnosed patients. A multidisciplinary decision was required before the initiation of hydroxychloroquine based on the COVID-19 treatment protocol.

**Conclusion:** COVID-19 has caused challenges and transformed access to health care. However, these limitations have provided opportunities for the health care system to adapt. Further, telemedicine has become a reliable platform for follow-up appointments that should be conducted by a multidisciplinary team including physicians, dieticians, and physical therapists.

## Introduction

Coronavirus disease 2019 (COVID-19) was first detected in Wuhan, China. Then, the virus rapidly spread worldwide. Hence, the World Health Organization declared a pandemic (1). As reported by Guo et al., COVID-19 is transmitted via respiratory droplets released into the air when an infected person coughs or sneezes (2). Patients with COVID-19 present with respiratory illness that is characterized by mild to severe symptoms. Older individuals and those with underlying conditions such as diabetes mellitus and hypertension have worse outcomes (3). Similar to global reports, some unpublished data have shown that pediatric patients (aged 0–18 years) had a lower proportion of COVID-19-related mortality than adult patients in Saudi Arabia. That is, only 5 of 35,222 pediatric patients die. There have been no known cases of COVID-19 patients with underlying neuromuscular disease admitted to any hospital.

The impact of COVID-19 pandemic on patients with neuromuscular conditions is significant and can range from severe respiratory complications to interference with their routine treatments, checks ups, and physical therapy (4).

Several papers have been published discussing care for patients during the pandemic and they mostly focused on maintaining minimum physical therapy and routine checkup (5). Other publications focused on the possible harmful pharmaceutical interactions of nutritional supplements in DMD (6).

Effective treatment strategies have not been established; thus, communities worldwide are advised to take non-pharmaceutical preventive measures to reduce COVID-19 transmission (7).

To inhibit the spread of the virus, the Centers for Disease Control and Prevention provided recommendations for the management of COVID-19 in healthcare and non-healthcare settings. The standard recommendations include good hygiene, social distancing, and use of a mask. Moreover, patients are encouraged to discuss an individualized treatment plan with their health care provider to prevent any risk of viral transmission (8).

Duchenne muscular dystrophy (DMD) is characterized by progressive muscle weakness and motor function deterioration, which lead to loss of ambulation by the age of 12–15 years DMD is attributed to an inherited x-linked mutation in the dystrophin gene that predominantly affects male individuals (9). The DMD gene plays a role in providing instructions to an essential cytoskeletal protein called dystrophin, which protects and stabilizes muscles (10). Becker muscular dystrophy is a milder condition that is characterized by x-linked muscular dystrophies. The age at onset is from 5 to 15 years, and it has varying symptoms that progress at a slower pace compared with those of DMD (11). Deregulation in gene expression or any mutation in the dystrophin gene ultimately leads to inadequate levels of dystrophin protein, thereby resulting in progressive muscle weakness and motor function deterioration overtime. Spontaneous mutations account for one-third of DMD cases (12). Most cases involve predominantly large deletions, as shown in the following mutational spectrum, which represents different types of mutations (13, 14): large deletions, 60–80%; large duplications, 7–11%; and non-sense mutations, ~10–15%.

### Prevalence and Symptoms of Duchenne Muscular Dystrophy

Information on the prevalence of DMD in the Middle East is relatively outdated. However, in Europe and North America, ~6 per 1,000,000 individuals are affected. Although there is no recent registry for patients diagnosed with DMD in Saudi Arabia, our experts confirmed a noticeable trend in which families are often found to have more than one child affected with DMD. It is not uncommon to have more than one male child affected with the condition in a given family considering that the average family size in the Middle East by itself is double if not triple compared with that of Western countries.

As muscle wasting occurs, patients experience muscle weakness primarily in the limb girdle muscle group with the lower limbs more severely affected than the upper limbs. Within the first 2 years of life, DMD patients exhibit mild gross motor developmental delays. Symptoms project in the form of recurring falls and struggle in climbing the stairs. Subsequently, muscle wasting symptoms gradually manifest until early 20 s, resulting in loss of ambulation and respiratory and cardiac dysfunction.

### Management of Duchenne Muscular Dystrophy

With consideration of different symptoms and possible complications, the standardization of treatment in patients with DMD is considered inadequate. For optimal treatment, a multidisciplinary approach that can fulfill the recommended standard of care is essential (9).

Duchenne muscular dystrophy treatments aim to control symptoms and prolong normal motor function to optimize quality of life. Glucocorticoids are routinely prescribed by healthcare providers as the standard treatment to improve muscle strength and function. However, the long-term use of steroids is not appropriate, and it indicates the need for alternative treatments such as dystrophin restoration therapies based on mutations in the DMD gene (9, 12, 15, 16). Some notable therapies include exon-skipping therapy for recovering the reading frame, read through therapy for non-sense mutation, and gene therapy using micro dystrophin, which is utilized in clinical trials. Thus far, there is no cure for DMD; however, advancements in the development of therapeutic strategies have shed a ray of hope for future therapies (13).

### Characteristics of Pediatric Duchenne Muscular Dystrophy Patients Admitted to the Intensive Care Unit/Hospital During the Coronavirus Disease 2019 Pandemic

Data on children with DMD are limited. However, studies have shown that the mortality rates and risks of acquiring COVID-19 are significantly lower in children than in adults. Pediatric cases account for 1–5% of all cases (17). Of 1,519 confirmed COVID-19 cases in a retrospective study in Saudi Arabia, 72 (4.8%) involved children. Adult and pediatric COVID-19 patients who were hospitalized accounted for 71.6% of all cases, and 4.7% required treatment in the intensive care unit, none of which had a pre-existing neuromuscular condition (18).

In this study, to standardize care and provide optimal treatment to DMD patients in Saudi Arabia during the COVID-19 pandemic, a panel of experts formed by the Saudi Pediatric Neurology Society (SPNS) consolidated recommendations for healthcare professionals and caregivers.

After reviewing literature related to the standard of care prior to and during the COVID-19 pandemic, the panel members compiled a list of statements to guide the virtual meeting. A consensus was reached and presented in this document after a series of discussions on the guidelines.

### Standard of Care for Duchenne Muscular Dystrophy Patients During the Coronavirus Disease 2019 Pandemic

COVID-19 is a new disease, and data that support the risk factors for severe diseases are limited. However, clinical experts believe that underlying comorbidities such as respiratory problems, cardiac dysfunction, and corticosteroid-induced immunosuppression are associated with a high risk of acquiring COVID-19 among DMD patients (19). Considering the vulnerability of high-risk groups, health care providers should be knowledgeable about COVID-19 mitigation strategies and should establish protocols to provide optimal care.

### Management of Respiratory Complications

Duchenne muscular dystrophy patients with respiratory complications should be timely referred to respiratory therapists to safely provide respiratory support. According to experts, the routine forced vital capacity test and respiratory function monitoring should be continued during the pandemic if necessary. Duchenne muscular dystrophy patients should seek medical attention if they suspect COVID-19. Considering the shortage in the supply of ventilators, patients are advised to bring their personal ventilation devices. Further, cough assist treatments should continue as scheduled, with consideration of safety measurements, to prevent mucosal trauma. Oxygen therapy is used to treat hypoxemia caused by hypoventilation. However, healthcare providers should be warned regarding the potential side effects of oxygenation without adequate ventilation to prevent complications. Thus, oxygen therapy is considered safe when combined with assisted ventilation and coughing. If tracheal intubation is required, it should be administered without neuromuscular blockade and depolarizing agents as they are relatively contraindicated in DMD patients (20). *Consensus recommendation:* Respiratory function monitoring and support should be continued during the pandemic if needed among DMD patients with respiratory complications.

### Management of Cardiovascular Complications

Cardiovascular disease in DMD patients is among the most common complications causing mortality. Thus, a strategy aiming for early diagnosis can lead to optimal treatment outcomes (20). Moreover, to minimize risks without compromising clinical outcomes, telemedicine is recommended as the main platform for follow-ups and for assessing patients who require in-person consultation based on precedency along with the consensus statement by the Cardiac Services Development Team of the Ministry of Health of Saudi Arabia (21).

Treatment for patients diagnosed with heart attack can be complex during current challenges in minimizing clinic visits. To treat the correct patient at the right time, clinical services should be prioritized by assessing the patient's medical history. Resource allocation must be evaluated based on the patients' need for assessment to minimize exposure and burden among patients and healthcare personnel without compromising clinical outcome.

- If a patient has been examined, echocardiogram (ECHO) and asymptomatic visit can be post-poned.
- If a patient is symptomatic and is on treatment, management should be continued until examined by a cardiologist.
- If a patient is symptomatic but is not receiving treatment, they should be urgently assessed by a cardiologist.

*Consensus recommendation*: During this pandemic, to reduce the risk of potential exposure without compromising clinical outcomes, cardiology follow-up service should be continued as a virtual visit if possible, and the decisions for in-person consultations with or without ECHO should be prioritized on a case-by-case basis by the cardiologist and neuromuscular specialist in accordance with the consensus statement by the Cardiac Services Development Team of the Ministry of Health of Saudi Arabia.

In DMD patients, the first-line pharmacological management or prophylaxis treatment for cardiomyopathy includes angiotensin-converting enzyme (ACE) inhibitors or angiotensin receptor blockers (ARBs). However, there has been a concern that patients who receive these medications are at high risk of severe acute respiratory syndrome coronavirus 2. However, this has been clarified by established organizations. The American Heart Association, American College of Cardiology, and Heart Failure Society of America have confirmed that individuals should continue to take these medications as the benefits outweigh the unknown risks of COVID-19 (20, 22). *Consensus recommendation:* Patients should continue taking ACE inhibitors or ARBs as treatment or prophylaxis for cardiomyopathy as their benefits outweigh the unknown risks of COVID-19.

### Rehabilitation Management

In accordance with the standard of care, in the rehabilitation management of DMD patients, the various needs and well-being of patients, caregivers, and staffs should be recognized and prioritized with the use of precautionary guidelines to make optimal decisions (12).

Patients are encouraged to continue physiotherapy as planned. To reduce transmission, physical therapists are advised to consider implementing tele rehabilitation strategies, and caregivers must provide support to patients under the therapist's supervision. Further, patients are recommended to increase physical activity in their home, and caregivers are encouraged to use additional rehabilitation considerations including intermittent bracing to help maintain activities of daily living and ambulation. *Consensus recommendation:* Rehabilitation management should be individualized, and the safety of patients, caregivers, and staffs must be prioritized.

Virtual clinics offer an opportunity for physiotherapists to continue providing high-quality care and meet patients' needs without interruption. Recent evidence has shown that transitioning into telehealth facilitates patient empowerment and enhances engagement (23). Virtual platforms can be leveraged by allowing patients to engage in online group classes, and high participation will encourage physical activity at home. *Consensus recommendation:* Virtual clinics can provide assurance that patients will still receive physiotherapy without interruption due to the current situation.

### Use of Glucocorticoids, Vitamin D Supplementation, and New Treatment Options

Several studies are currently investigating the safety of commonly used medications in response to COVID-19. Research has been conducted after speculations over the use of corticosteroids, which is a drug commonly used by DMD patients. Glucocorticoids have long-term benefits as treatment for DMD, and physicians are advised to continue prescribing the medication as a standard of care among patients who are either receiving prednisone (0.75 mg/kg/day) or deflazacort (0.90 mg/kg/day) (12). Thus far, there is no evidence showing that corticosteroid therapy at a lower dose will lead to a lower risk of viral infection. It is worth noting that several studies suggest that patients with chronic lung diseases, including asthma or rheumatological disorders who are prescribed long-term glucocorticoid therapy (GC) account for a smaller percentage of patients diagnosed with severe COVID-19 (24). Although these findings require further validation as they may be ascribed to confounding factors and reporting bias, GC's should continue to be prescribed. Thus, experts advise physicians to prepare themselves in adjusting dosage as a precautionary measure if the patient's condition exacerbates. Medication regimens can be modified using stress dose during acute illness and substituting the administration of a high dose from weekends to daily (25). *Consensus recommendation:* Patients should continue glucocorticoid treatment; however, dose adjustment is recommended if the condition exacerbates.

It is important to consider the adjuvant use of Vitamin D supplements with steroids. One study has reported that up to 25% of DMD patients suffer from vitamin D deficiency due to various reasons (26, 27). Vitamin D has many proven benefits in fighting viral infections, including influenza and HIV. Moreover, its effect against COVID-19 has been postulated but more clinical evidence is required (28). *Consensus recommendation:* DMD patients, especially those on steroids should be prescribed with vitamin D supplements.

New gene correction therapies for DMD including ataluren, which targets non-sense mutations, have recently been approved. Patients should continue using ataluren as it is administered orally. However, safety rules dictate that biochemical parameters should continuously be monitored.

There are concerns regarding the safety of office-administered DMD therapies such as the newly approved antisense oligonucleotides that are administered via intravenous (IV) infusion; eteplirsen, which binds to exon 51; and goldcrest and viltoarsen, which bind to exon 53. The pandemic has emphasized the need for home infusion to address safety concerns in vulnerable populations. However, if not applicable, experts advocate for treatment continuation in clinical settings while adhering to safety protocols. In patients who have not started on the IV treatment, therapy should be delayed to prevent infections. *Consensus recommendation: IV* Treatment should be post-poned in newly diagnosed patients to prevent hospitals visits and acquiring infections. In patients who are already receiving IV infusion, all precautionary measures and safety protocols should be followed. However, home infusion is recommended if applicable. While Ataluren, an orally administered treatment, should be continued.

There are several controversies regarding the use of hydroxychloroquine (HCQ) for the treatment of COVID-19. Reports have shown that this drug may be beneficial; however, it should be used with caution particularly in high-risk patients. Despite obtaining positive outcomes, HCQ poses deleterious effects in some DMD patients such as those with cardiac arrhythmia and vacuolar myopathy. Due to the possible risk of complications and uncertainty in benefits, experts recommend discussing the decision with the primary neuromuscular and cardiology specialists on an individual basis (29–32). *Consensus recommendation:* Due to potential risks and uncertainty in the benefits of HCQ for the treatment of COVID-19, the decision must be discussed with the primary neuromuscular and cardiology specialists on an individual basis among DMD patients.

These are general and unofficial recommendations (Table 1). Different scenarios should be evaluated by neuromuscular specialists along with the patient to provide timely treatment.

**Table 1:** Summary of recommendations for the standard of care in DMD patients during the COVID-19 pandemic.

**Area**	**Recommendations**
Respiratory complications	- Respiratory function monitoring and support should be continued as needed.
Cardiovascular complications	- Telehealth should primarily be used for follow-up appointments. Considering the risk of viral infection, ECHO does not seem appropriate for all patients, and it may require a case-by-case evaluation. Patients should continue taking ACE inhibitors or ARBs as treatment or prophylaxis for cardiomyopathy.
Rehabilitation	- Rehabilitation management should be individualized, and the safety of patients, caregivers, and staff should be prioritized. Further, physiotherapy should be continued without interruption in virtual clinics.
Medications	- Patients should continue their current medication (glucocorticoid or gene therapy). - Due to the immunosuppressive effects of steroids, dose adjustment may be required if the condition exacerbates. - Gene therapy should be post-poned in newly diagnosed patients. - A multidisciplinary decision on an individual basis is recommended before initiating treatment with HCQ.

*DMD, Duchenne muscular dystrophy; ACE, angiotensin-converting enzyme; ARBs, angiotensin receptor blockers; ECHO, echocardiogram; HCQ, hydroxychloroquine*.

## Conclusion

COVID-19 has brought challenges and transformed access to health care. However, these limitations have provided opportunities for the health care system to adapt. Telemedicine has become a reliable platform for follow-up appointments that should be conducted by a multidisciplinary team including physicians, dieticians, and physical therapists.

## Data Availability Statement

The original contributions generated in the study are included in the article/supplementary material, further inquiries can be directed to the corresponding author.

## Author Contributions

AB directed the project, led the discussion and summarized the consensus. The rest of the authors contributed equally in data collection, sharing practice, discussing management, and writing the manuscript. All authors contributed to the article and approved the submitted version.

## Conflict of Interest

The authors declare that the research was conducted in the absence of any commercial or financial relationships that could be construed as a potential conflict of interest.
